# Spina Bifida

**Published:** 1893-07

**Authors:** James Kennedy

**Affiliations:** San Antonio


For Texas Medical Journal.

### SPINA BIFIDA, WITH A REPORT OF A CASE—OPERATION—RECOVERY.

BY JAMES KENNEDY, M. D., PH. G., SAN ANTONIO.

Read before the San Antonio Medical and Surgical Society, April, 1893.

MR. PRESIDENT AND GENTLEMEN:—If you will kindly bear with me for a few minutes, I will invite your attention to the subject of Spina Bifida.

Spina Bifida is a tumor which forms upon any portion of the spine. Its coverings consist of the tegumentary structures and the membranes of the cord.

The contents of the tumor varies,—sometimes it contains nerve filaments, nerves, or even a portion of the cord itself, but a constant and essential constituent is the cerebro-spinal fluid.

With reference to the pathology of Spina Bifida, two theories have been advanced,—one is that an early dropsy of the membranes of the cord takes place and prevents ossification of the lamina; another is, that defective ossification is entirely responsible for the deformity. It seems to me that both theories are tenable, and it may be that an early dropsy of the cord membranes produces a Spina Bifida in some cases, while defective ossification brings about the same result in others.

As to the relative frequency of occurrence of this phenomenon statistics seem to show that it has been found about twenty-two times in a little over twenty-two thousand births, or about one per thousand births.

With reference to the diagnosis of Spina Bifida the following points are to be born in mind: The tumor is always situated in the median line and may form in any region of the spine. It has been discovered that a Spina Bifida may form upon the anterior side of the column, and indeed there seems to be no good reason why an accidental failure of the development of bone should not occur as well on one side of the column as the other. The size of the tumor varies from a small sized hen's egg to a fœtal skull, or even larger. It is almost always congenital, although in a few well authenticated cases the tumor made its appearance sometime after birth. The tumor is slightly reducible by pressure, forcing the fluid back into the sub-arachnoidean space.

It occurs most commonly in the lumbo-sacral region. About six-sevenths of all tumors of this character are found in this region; the other seventh is found in the cervical or servico-dorsal. They are very rarely found in the mid-dorsal region.

The tumor may be either pedunculated or sessile.

With reference to the treatment of Spina Bifida we are still very much in the dark, and we have almost as many diverse opinions as we have authorities on this subject.

There are some who favor compression of the sac in order to accomplish absorption of its contents; others favor evacuation by puncture, and there are those who favor the injection of iodine solution, (Morton's) either with or without the withdrawal of a portion of its contents, and again there are those who believe in the ligature, whilst there are still others who believe in the wholesomeness of the knife in the treatment of this malady.

When we consider the variability of the contents of the tumor, it is not to be wondered at that opinions are so diverse as regards the treatment of this affection, nor is it remarkable that such different results have been obtained by different operators. If the tumor contains nerves of considerable size or a portion of the cord, as is sometimes the case, it is not to be wondered at that the knife and ligature have failed to cure, or have even killed in a certain considerable number of cases; nor does it require a very great stretch of the imagination to appreciate how sudden collapse may occur from the rapid emptying of the tumor, especially if it be situated high up on the spinal cord.

But when a tumor is situated on the lower portion of the column and contains only cerebro-spinal fluid, nerves and cord being absent, it seems to me that extirpation is the most rational method of procedure. Even if it is situated high up, if the tumor is tied off by means of a rubber ligature before dissection, and then carefully emptied, if no nerves be present, it would seem to me a moderately safe operation.

The case which I am about to report, was a patient of Dr. H. J. Trollinger, of this city, and from whom I obtained the following history:

CASE.

"A negro woman, about thirty-five years of age, and the mother of three children. Tumor about as large as the head of a six months child. History, as given by patient, says that up to six years ago the tumor was about as large as a hen's egg; since then it has grown steadily. Fourteen months ago it began to exude its fluid contents through the pores of the skin. This lasted until the sac was entirely shrunken and flabby; then it began to refill and enlarge. Patient has for years (three or four) complained of a dragging sensation in back of head and neck; a tired feeling of eyes, pain throughout back on walking, and pain in lower limbs when walking."

At the time I saw the case, the tumor was situated at the junction of the fifth lumbar vertebra with the sacrum; it was sessile and about the size of a good sized infant's head. It was fluctuating in character and was very slightly reducible. The integument covering the mass was very thin and tense, and contained cicatrices from previous ulceration.

I was invited by Dr. Trollinger to operate, and with the very able assistance of himself and Dr. Russell Caffery, I proceeded to remove the tumor in the following manner:

An elliptical incision, which included the greater portion of the thinned integument, was made, and the remainder of the skin, adherent to the tumor, was dissected away. The mass now being freed down to the point of attachment to the cord, it was opened by incision, and slowly emptied.

Its interior was next examined, and was found to consist of a white, glistening sac, with a bluish tinge. Neither nerves nor cord were contained in it. On examination, a lumen of nearly a half inch, was found at the base of the tumor, and it was clear that it communicated with the cord. The sac was ligated at its point of origin from the cord, well within the spinal canal. The remaining portion of the thinned integument was cut away, on account of its comparatively lifeless character, as I felt sure it would slough. Its removal left a very large area of denudation, which was closed in by the approximation of the cut surfaces. Silver wire was used for deep suturing, and silk was used superficially.

The subsequent history of the case has been furnished me by Dr. Trollinger, and is as follows:

''Patient reacted well from shock of operation, which seemed relatively slight. For first thirty-six hours there was but little elevation of temperature; later, however (fourth day), the temperature was 99.5° F., and rose steadily to 103.4° F., where it remained for about six days, when it began to subside, and became normal on the tenth day, and so remained.''

This temperature was evidently septic, because some suppuration occured in the deeper structures. The wound was imperfectly drained by the strips of antiseptic gauze, which we had left in the wound for that purpose. The skin wound united by first intention. As soon as the wound was flushed, the temperature began to subside, and after repeated flushings, became normal.

I regard this case as being of interest, on account of the comparatively small number of cases of this affection who live to the age of thirty-five years, and on account of the successful results following the operation. It is now almost eight weeks since the operation, and the patient has entirely recovered from its effects. The operation was done under extremely unfavorable circumstances, and even a moderate approach to asepsis was impossible to obtain.

